# Correction: Graphene oxide-based silsesquioxane-crosslinked networks – synthesis and rheological behavior

**DOI:** 10.1039/d4ra90154a

**Published:** 2025-01-29

**Authors:** Mina Namvari, Lei Du, Florian J. Stadler

**Affiliations:** a College of Materials Science and Engineering, Shenzhen Key Laboratory of Polymer Science and Technology, Guangdong Research Center for Interfacial Engineering of Functional Materials, Nanshan District Key Lab for Biopolymers and Safety Evaluation, Shenzhen University Shenzhen 518060 PR China fjstadler@szu.edu.cn +86-0755-26536239 +86-0755-26538236; b Key Laboratory of Optoelectronic Devices and System of Ministry of Education and Guangdong Province, College of Optoelectronic Engineering, Shenzhen University Shenzhen People's Republic of China

## Abstract

Correction for ‘Graphene oxide-based silsesquioxane-crosslinked networks – synthesis and rheological behavior’ by Mina Namvari *et al.*, *RSC Adv.*, 2017, **7**, 21531–21540, https://doi.org/10.1039/C7RA02764H.

The authors regret that the Raman data in Fig. 3 cannot be relied upon. They found after publishing the data that the background scatter compensation routine of the Raman measurement was omitted by the operator but that it does not affect the peaks that are used to prove the successful synthesis.

An independent expert has concluded that the other data does support the conclusions that the material has been synthesised.

The Royal Society of Chemistry apologises for these errors and any consequent inconvenience to authors and readers.

